# Influence of reduction accuracy in lateral tibial plateau fractures on intra-articular friction – a biomechanical study

**DOI:** 10.1186/s12891-019-3020-3

**Published:** 2020-01-11

**Authors:** Christian Walter, Alexander Beck, Christopher Jacob, Ulf Krister Hofmann, Ulrich Stöckle, Fabian Stuby

**Affiliations:** 1 University Hospital Tübingen , Hoppe Seyler Str. 3, 72076 Tübingen, Germany; 2 Orthopedic Biomechanics Laboratory, University Hospital Tübingen, Hoppe Seyler Str. 3, 72076 Tübingen, Germany; 3 Charité – Berlin, Augustenburger Platz 1, 13353 Berlin, Germany; 4 BG Murnau, Prof.-Küntscher-Straße 8, 82418 Murnau, Germany

**Keywords:** Tibial head fracture, Trauma surgery, Friction, Knee joint, Dissipated energy, Biomechanics, Reduction

## Abstract

**Background:**

Lateral tibial split fractures (LTSF) usually require surgical therapy with screw or plate osteosynthesis. Excellent anatomical reduction of the fracture is thereby essential to avoid post-traumatic osteoarthritis. In clinical practice, a gap and step of 2 mm have been propagated as maximum tolerable limit. To date, biomechanical studies regarding tibial fractures have been limited to pressure measurement, but the relationship between dissipated energy (DE) as a friction parameter and reduction accuracy in LTSF has not been investigated. In past experiments, we developed a new method to measure DE in ovine knee joints. To determine weather non-anatomical fracture reduction with lateral gap or vertical step condition leads to relevant changes in DE in the human knee joint, we tested the applicability of the new method on human LTSFs and investigated whether the current limit of 2 mm gap and step is durable from a biomechanical point of view.

**Methods:**

Seven right human, native knee joint specimens were cyclically moved under 400 N axial load using a robotic system. During the cyclic motion, the flexion angle and the respective torque were recorded and the DE was calculated. First, DE was measured after an anterolateral approach had been performed (condition “native”). Then a LTSF was set with a chisel, reduced anatomically, fixed with two set screws and DE was measured (“even”). DE of further reductions was then measured with gaps of 1 mm and 2 mm, and a 2 mm step down or a 2 mm step up was measured.

**Results:**

We successfully established a measurement protocol for DE in human knee joints with LTSF. While gaps led to small though statistically significant increase (1 mm gap:ΔDE compared with native = 0.030 J/cycle, (+ 21%), *p* = 0.02; 2 mm gap:ΔDE = 0.032 J/cycle, (+ 22%), *p* = 0.009), this increase almost doubled when reducing in a step-down condition (ΔDE = 0.058 J/cycle, (+ 56%), *p* = 0.042) and even tripled in the step-up condition (ΔDE = 0.097 J/cycle, (+ 94%), *p* = 0.004).

**Conclusions:**

Based on our biomechanical findings, we suggest avoiding step conditions in the daily work in the operating theatre. Gap conditions can be handled a bit more generously.

## Introduction

Lateral tibial split fractures (LTSF) occur mainly in young patients in the context of high-energy traumas (mostly road accidents) and are often serious injuries. Surgical therapy is required in most cases and two groups of surgical treatment options are available: State of the art treatment is open reduction and internal fixation. This is usually achieved by performing an anterolateral approach combined with an angle-stable plate osteosynthesis or screws. In selected cases a minimally invasive procedure with screws in conventional two-screw osteosynthesis technique or jail-technique can be performed [1]. Reduction control can thereby be done fluoroscopically or arthroscopically [2]. Depending on the fracture classification, the surgeon selects the appropriate procedure.

One prior aim of surgery is the anatomical reduction of the fracture [3]. Non-anatomical reduction with gap- or step-condition leads to increased cartilage wear and in the further progression to cartilage damage and post-traumatic osteoarthritis. In daily practice, anatomical reduction can be challenging and is not achieved in all cases [4], especially in complex fractures or large soft tissue damage.

In the last decades various works had been published which tried to address the question, how good reduction of the fragments needs to be from a clinical point of view. Older data from Blokker et al. described a residual step of less than 5 mm on the weight-bearing area as a satisfactory result [4]. Others tolerated tibia plateau widening up to 10 mm [5]. In the last 10 years several studies were published which propagate a gap and a step of maximum 2 mm as tolerable limit [6–8]. Interestingly, increased histological cartilage damage was already found by Goetz et al. in an animal study with minipigs after reduction with a 2 mm step-off, compared with anatomical reconstruction in distal tibia fractures [9]. Defining the maximum of acceptable step-off therefore seems to remain an unsolved problem.

To date, biomechanical studies regarding tibial fractures have been limited to pressure measurements [10, 11] or histological staining [9, 11]. The effect of malalignment after reduction on actual joint friction in tibial plateau fractures has so far not yet been investigated.

We had previously demonstrated in a sheep model, that different cartilage defect levels can be characterized by measuring the dissipated energy (DE) using a 6 degree of freedom robotic system [12]. Advantage of this experimental system is that it allows a friction calculation in a whole knee joint with the total joint surface and all friction properties in their individual joint environment including the ligaments and the synovial fluid [13]. Previous studies in our lab had established the DE as a suitable system to describe the friction properties in the knee joint [13, 14].

Aim of the present study was to transfer our sheep model for characterizing cartilage defects to human joints with split fractures of the lateral tibial plateau. Furthermore we wanted to investigate the effect of different types of reduction with screw osteosynthesis using DE as experimental outcome parameter. Based on the current recommendations, we measured DE for anatomical reduction and malreduction with a horizontal gap of 1 or 2 mm and a vertical step of plus and minus 2 mm. From previous data in the sheep model with osteochondral transplant positioning (unpublished data) we hypothesized that especially the vertical step-up reduction would lead to increased friction in the joint.

## Methods and material

### Specimens

Seven right human, native and non-fixed knee specimens with soft tissue and skin were used for the experiments. These were stored at − 18 °C and thawed for the investigations slowly during 12 h at room temperature. The specimens were administered via Science Care (SCIENCE CARE, Arizona Headquarters, 21,410 N. 19th Ave., Suite 126, Phoenix, AZ 85027, United States, Fax: 602.331.4344).

### Robot

A robotic 6-degree-of-freedom setup (KUKA KR 60–3 robot, Augsburg, Germany; reproducibility: ±0.06 mm) including a universal force/torque sensor (ATI UFS: Theta SI1000–120; resolution: 0.25 N and 0.025 Nm) was used to perform axially loaded knee flexion.

### Screws

4.5 Mm cannulated, self drilling screws with long thread, available in lengths from 20 to 80 mm (article number X14.620–672, Synthes GmbH, Oberdorf, Switzerland) were used for screw osteosynthesis

### Experimental procedure

**Fig. 1:**
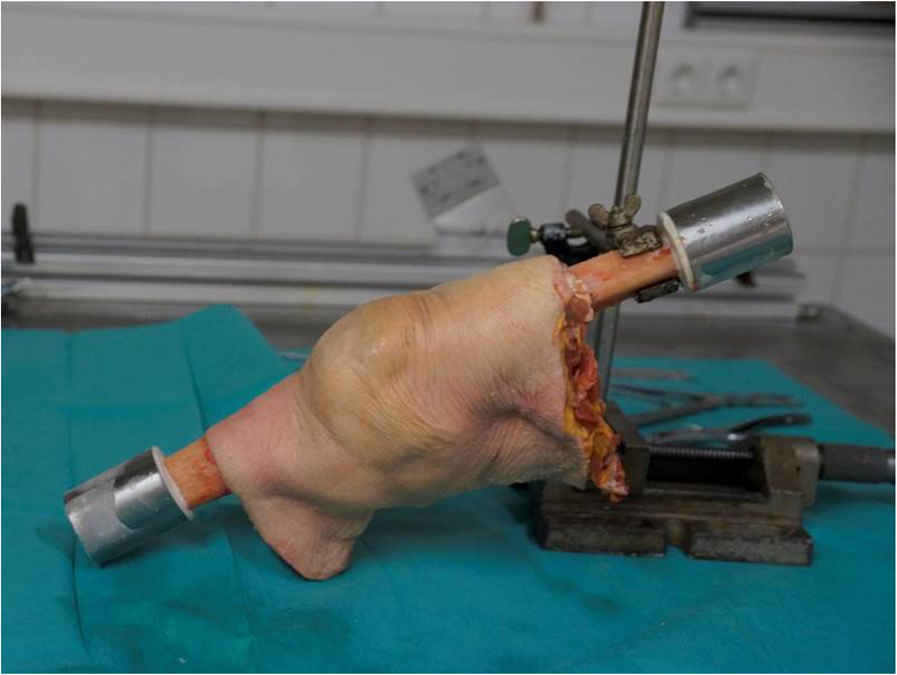
Specimen after preparation and fixation with intact skin and soft tissue. Tibia and femur are embedded in resin and covered with an aluminum cylinder. The tibia was resected at the level of the lower soft tissue border

Osteotomies of the femur and tibia were performed 25 cm above / below the joint space. At a distance of about 8 cm distal / proximal to the osteotomy, the bones were completely cleaned of periosteum and embedded in 2-component resin (RenCast© FC 53 isocyanate/FC 53 polyol, Gößl & Pfaff GmbH, Karlskron, Germany) (see Fig. 1). The settings of the robot used for previous studies with non human specimens [13] were adapted to the larger human knee joints. Fixation within aluminum cylinders in the robot, axes definition and recording of the passive path remained unchanged as described in previous studies [13, 14].

First, an antero-lateral approach to the knee joint was performed as is usually done in lateral tibial fractures, to prevent access-related falsification of the measured values [15]. Then the approach was closed by suture to prevent dehydration during the measurement. In the next step recording of the individual passive flexion path was performed [16, 17], which is required for further measurements. Passive path is described as a flexion way of minimal resistance unique for each knee joint [16].

Axial compressive load of 400 N (approximate half of the body weight) was applied to the femur during the recording (higher loads leads to specimen failure). Subsequently, the first measurement of the DE was performed on the ‘native’ knee, traversing the measured path and plotting friction torque against the flexion angle. From the area enclosed within the hysteresis curve the DE can be calculated.

Second, we reopened the joint and prepared the lateral soft tissues in order to induce a lateral tibial split fracture (AO: 41-B1, Schatzker Typ I [18], Tscherne u. Lobenhofer P1 [19]) After pre-drilling, two screws were inserted from the lateral side into the intact bone and were removed after fluoroscopic control. The resulting screw holes later enabled optimal fracture reduction. The fracture zone was marked with Kirschner wires exactly in the center of the lateral compartment under fluoroscopic control, because a too lateral fracture planning resulted in a covering of the fracture by the meniscus and too medial approach resulted in complex tibial fractures in preliminary experiments.

Subsequently, the bone fracture was induced using a chisel: Initially, the chisel was inserted in a sagittal plane into the lateral tibia without affecting the lateral cortical bone or the joint. Rotation of the chisel then resulted in fracture of the chondral and subchondral structures, as well as the lateral cortical bone. Great care was taken not to touch the surface structures with the chisel to ensure a fracture line as realistic as possible and to avoid intra-articular injuries with the chisel, which might later influence the measured DE.

**Fig. 2:**
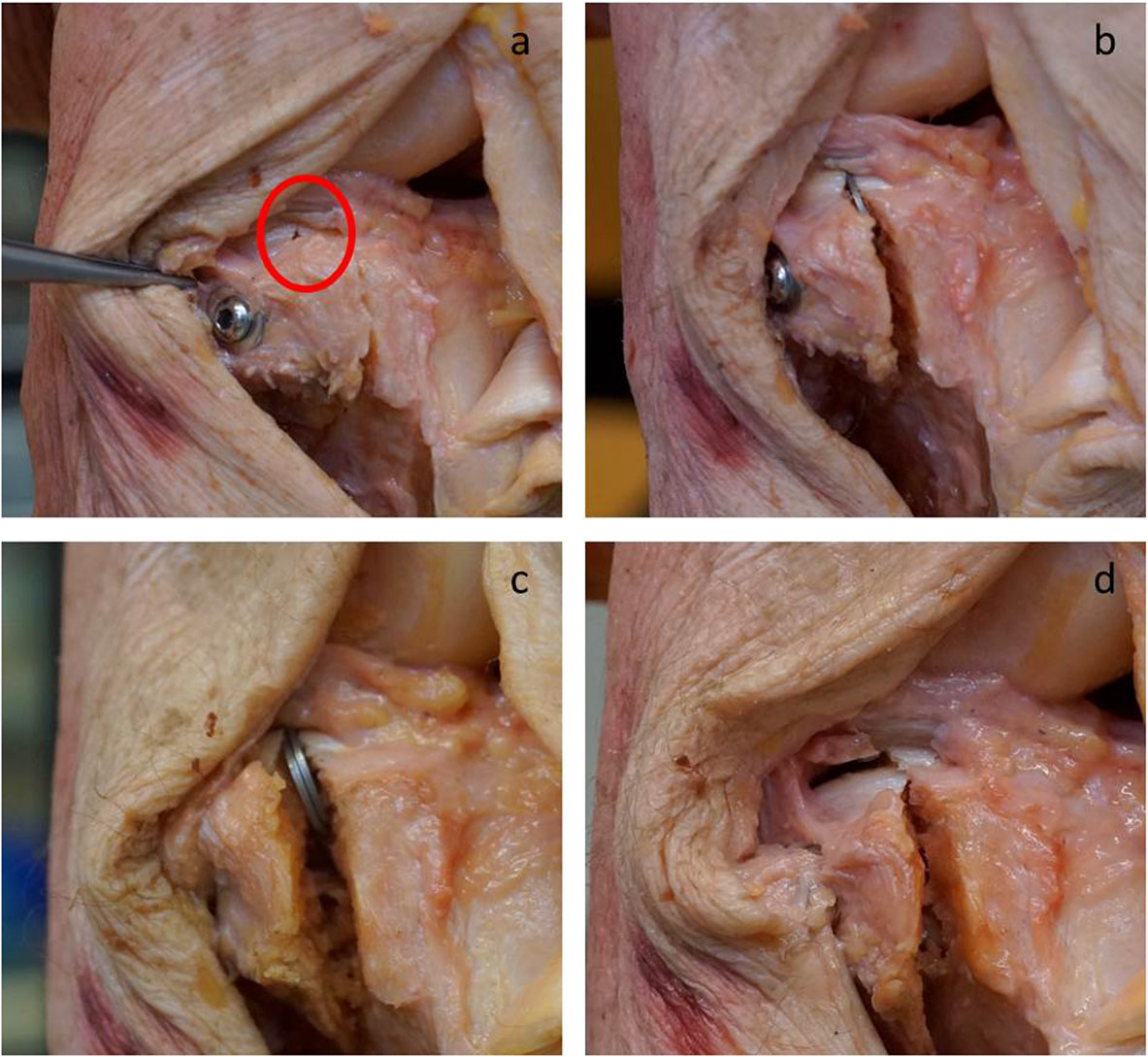
Representation of the different conditions. Correct reduction (even) with 2 parallel set screws (**a**). Gap of 1 mm with a 1 mm washer directly under the cartilage surface (**b**), of 2 mm with two 1 mm washers (**c**), and a 2 mm step down with a split below the meniscus (**d**)

Third, the fracture was reduced anatomically and the screws inserted into the existing holes (see Fig. 2a). The DE could thus in a next step be measured in the condition we labelled ‘even’. Thereafter, the fracture gap was simulated with two 1 mm washers, which were inserted anteriorly and posteriorly into the fracture gap (see Fig. 2b) and the DE measured for this condition called ‘1 mm gap’. To enlarge the fracture gap, a further washer was added in each side (‘2 mm gap’) and again DE was measured (see Fig. 2c).

Finally, we removed the screws and enlarged the screw holes in the lateral fragment in an oblong direction to obtain a displacement distance of the fragment of 4 mm. Subsequently, the fragment was fixed again in the ‘step up’ / ‘step down’ condition with the screws and washers and we carried out the corresponding measurement (see Fig. 2d). During the whole experiment, for each measurement, the approach was closed again with sutures to prevent the dehydration of the specimen and to preserve the synovial fluid.

### Data analysis

The knee flexion movement varies for ±10° about the central flexion angle of approximately 60°. The axis of rotation varies with the vertical load, the reduction condition and with the flexion angle. The intersegmental force and moment are functionally meaningful if they are defined at the “joint center” that lies on the axis of rotation [20]. Therefore the screw axis identification method was used to determine the instantaneous screw axis parameter for each displacement  from position  to  using the robot coordinates of the tool center point. The point on the helical axis and the unit vector of the helical axis with reference to *P*_*n*_ are then transformed back to Cartesian base coordinates. This is done for a complete cycle in flexion angle steps of 1°. The median helical axis defines the lateral axis of the new reference system. The wrench vectors consisting of the forces and moments at the base frame are transformed to the new reference frame. The calculations were done using an open source robotics toolbox for MATLAB [21].

**Fig. 3:**
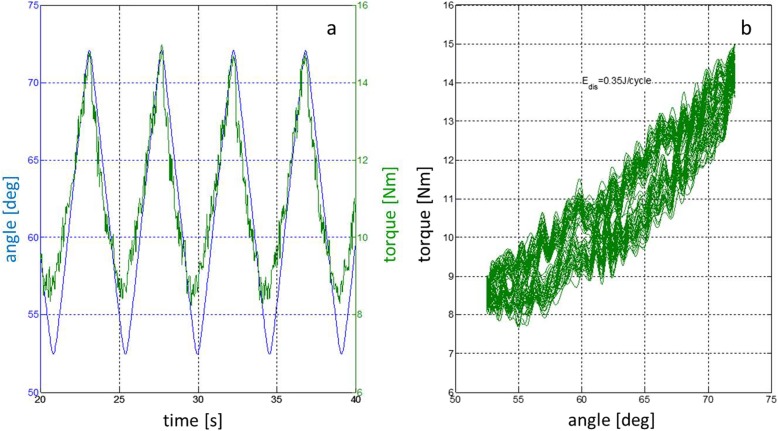
Measurement registration of the experimental set-up. Representative time plot for knee motion during several measurement cycles with flexion angle [degree] (blue) and resulting torque force [Nm] (green] (**a**) and the corresponding hysteresis curve (**b**) for the condition ´step up`. The dissipated energy is represented by the area enclosed within the hysteresis curve

A low pass filter was used to plot the torque values of the force / torque sensor. Figure 3 shows the torque-time diagram and the corresponding hysteresis curve for the condition ‘step up’. The DE is represented by the area within the hysteresis curve. The DE for one cycle is calculated with Formula. The measurement was done for 40 cycles. The first two cycles were omitted in the calculation of the mean dissipated energy per cycle. The integral was calculated using the Simpson integration rule from unfiltered torque values since the white noise compensates during integration and the median DE of 38 cycles was calculated for further analysis using SPSS-Statistics (IBM, Version 25.0.0.1).

Formula

$$ E(dis)={\oint}_{\varphi }M\  d\varphi $$

E (dis): dissipated energy.

M: torque.

φ: flexion angle.

Formula 1: Calculation for the dissipated energy

## Results

All experimental conditions could be measured in four specimens. In the fifth specimen screw failure occurred during the measurement of the condition ‘step down’. For this specimen only the conditions ‘native’, ‘even’, ‘1 mm gap’ and ‘2 mm gap’ are available. Therefore we measured in the sixth knee only the conditions ‘native’, ‘even’, ‘2 mm step down’ and ‘2 mm step up ‘to get a complete dataset for five specimens. In one cadaveric knee we accidentally induced a complex proximal tibial fracture during the first measurement cycles with the axial force of the robot so that this sample was completely excluded.

First, we investigated if the measurement of the DE is also possible in the human joint under fracture conditions. The measurement stability of the system was examined by comparing the values obtained from the native knees (*M* = 0.140 J/cycle, *SD* = 0.062, *n* = 5) with those in the condition ‘even’ (*M* = 0.165, *SD* = 0.067, *n* = 5) after the fracture had been induced and anatomical reduction been performed, with both conditions showing similar results (*p* > 0.999). Therefore we do not consider interfering factors, such as microfragments or dehydration caused by the procedure to be of relevance.

Second, we investigated if a horizontal gap in the tibial plateau after fracture reduction leads to a change in DE in the knee joint (Table 1).

**Table 1:** Values for gap (a) and step (b) conditions. Different values for ‘native’ and ‘even’ in (a) and (b) resulting from different datasets caused by specimen failure

	N	Mean	Standard deviation	Minimum	Maximum	percentile		
						25.	50. (Median)	75.
a								
native	5	0.1207	0.06169	0.02	0.18	0.0629	0.1399	0.1691
even	5	0.1339	0.06748	0.02	0.19	0.0693	0.1648	0.1830
gap_1mm	5	0.1660	0.09830	0.03	0.29	0.0787	0.1703	0.2510
gap_2mm	5	0.1587	0.08262	0.03	0.23	0.0797	0.1720	0.2310
b								
native	5	0.1021	0.06821	0.02	0.18	0.0345	0.1033	0.1691
even	5	0.1143	0.07035	0.02	0.19	0.0458	0.1141	0.1830
2 mm step down	5	0.1471	0.08844	0.04	0.25	0.0571	0.1612	0.2300
2 mm step up	5	0.1897	0.12590	0.04	0.35	0.0659	0.1999	0.3084

**Fig. 4:**
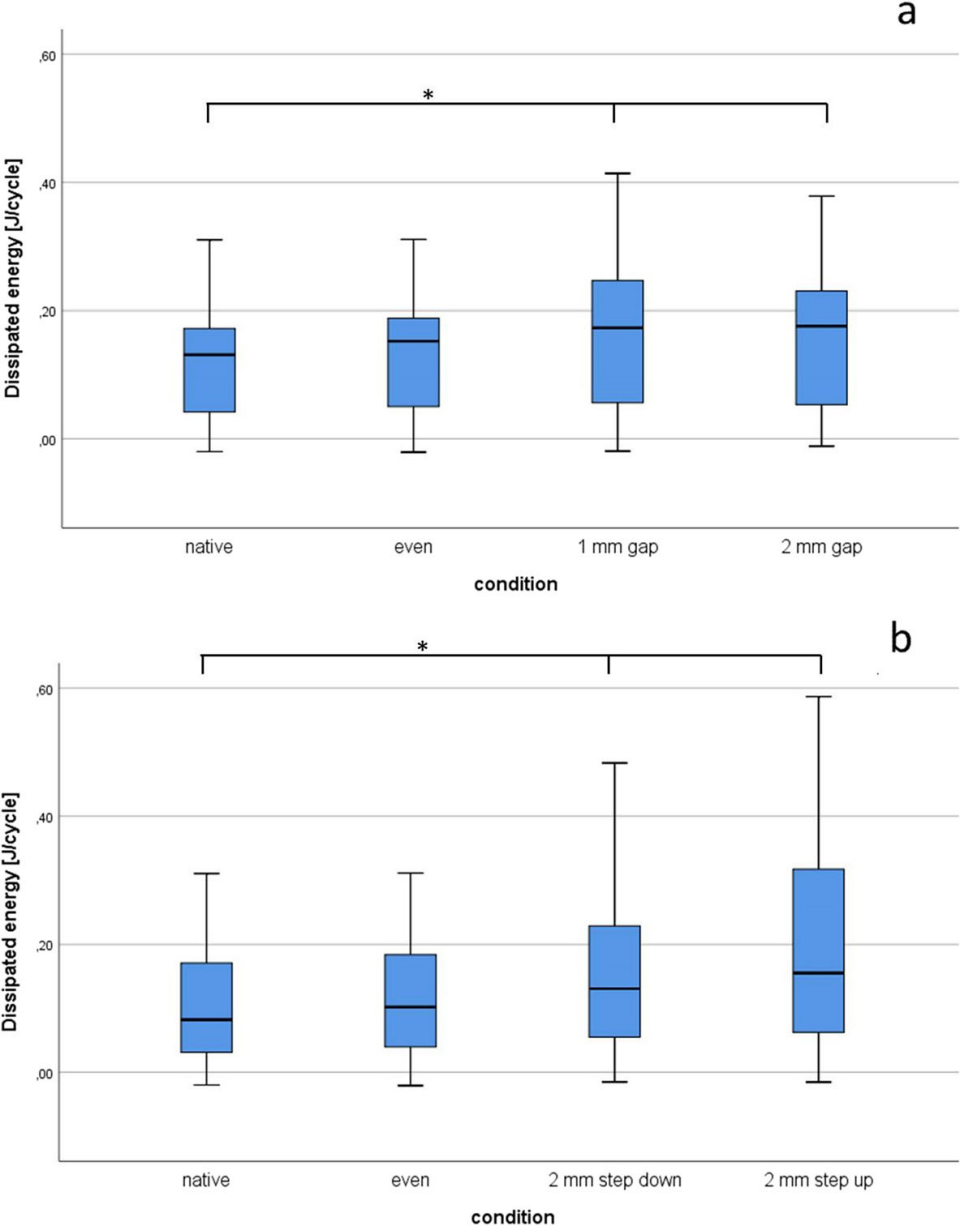
Dissipated energy (DE) for the different measurement conditions**.** Boxplots displaying the DE **[**J/Cycle**]** measured in the native condition and lateral tibial plateau fractures reduced with different horizontal gaps (**a**), and vertical fracture displacement (**b**). While no major difference can be seen between a horizontal one and two mm gap, DE strongly increases in the step-down reduction and is threefold increased in the step-up condition. * denotes a significant difference (*p* < 0.05) after Friedman’s group test with Bonferroni correction

Therefore, as described above, DE was measured for the conditions ‘native’, ‘even’, ‘1 mm gap’ and ‘2 mm gap’ (Fig. 4a). The DE of the four defect grades were not normally distributed, as assessed by the Shapiro-Wilk test (*p* < 0.001). To compare treatment effects DE data were analysed with the nonparametric Friedman’s test [14]. The four conditions (native, even, 1 mm gap, 2 mm gap) showed significant changes in DE (Friedman test χ^2^(3) = 13.560, *p* = 0.004, *n* = 5). Subsequently the results of Friedman’s test underwent Dunn’s pairwise post hoc tests and Bonferroni adjustments of the *p*-values. The level of significance was chosen as *p* = 0.05. Significant differences were found between ‘native’ and ‘1 mm gap’ (*p* = 0.02; Δ DE = 0.030 J/cycle), as well as ‘native’ and ‘2 mm gap’ (*p* = 0.009; Δ DE = 0.032 J/cycle). The effect sizes (r) were calculated using the formula *r* = z/√n (z = test statistic, *n* = number of pairs). A strong effect (r > 0.50) was found for ‘native’ vs. ‘1 mm gap’ (*r* = 0.98) and ‘native’ vs. ‘2 mm gap’ (*r* = 1.06). Interestingly a higher gap size did not result in also higher DE as measured in the conditions ‘1 mm gap’ (M = 0.170 J/cycle, SD = 0.983) and ‘2 mm gap’ (M = 0.172 J/cycle, SD = 0.826) (*p* > 0.999).

Finally we asked if a vertical step in the tibial plateau after fracture reduction leads to a change in DE in the knee joint. The conditions ‘2 mm step-up’ (Δ DE compared with ‘native’ 0.097 J/cycle) and ‘2 mm step-down’ (Δ DE compared with ‘native’ 0.058 J/cycle) showed significant changes in DE when compared to the conditions ‘native’ and ‘even’ (Friedman test χ^2^(3) = 14.04, *p* = 0.003, *n* = 5). In Dunn-Bonferroni post-hoc tests significant differences with strong effect sizes were observed between ‘native’ and ‘2 mm step-down’ (*p* = 0.042; *r* = 0.90) and ‘2 mm step up’ (*p* = 0.004; *r* = 1.14) after Bonferroni adjustments of the *p*-values. No significant differences were found between any other conditions.

## Discussion

The main function of synovial fluid is the lubrication of the cartilage in synovial joints [22, 23]. Especially the lubricant molecules hyaluronan and proteoglycan 4 [24] are indispensable to reduce the friction in the joint. Dehydration during the experiment leads to loss of synovial fluid which in turn could lead to an increase of DE during the experiment. Therefore, we took great efforts to preserve the synovial fluid and reclosed the approach by suture after every surgical step. In the comparison of the original ‘native’ condition and the ‘even’ condition of the anatomical reduction situation no significant changes in DE could be detected, but rather a comparabel level of DE was measured. Our results thus confirmed that the sheep model for characterizing cartilage defects can be translated to human knee joints with lateral tibial split fractures. No major difficulties could be detected during the technical adjustment.

In a previous study we had detected a significant increase of DE in only small cartilage lesions [12]. Isaac et al. also described chondrocyte damage after a single impact without fracture in rabbit tibiofemoral joints [11]. These examples document the sensitivity of cartilage in high energy overload. Consequently, when performing the fracture, we avoided direct contact of the chisel with the cartilage surface. As mentioned before, we found no significant difference in the DE before and after fracture.

In biomechanical testing, the described DE method has three main advantages: First we receive a natural testing environment by using the whole joint with all ligaments, menisci and the synovial fluid [13]. Other biomechanical studies concerning this topic are working without soft tissue [25] or with synthetic bone [26]. Second we can adapt robotic motion to a realistic situation by recording the passive path [14]. This is an advantage compared with other studies working with different flexion states (0° and 30°) [10]. Third, with measuring DE we can determine a friction parameter, different from studies measuring pressure parameters [10, 27, 28]. Friction is directly associated with wear of the cartilage, as Jay et al. could demonstrate in Lubricin-lacking mice [29]. Therefore the DE might illustrate a more realistic view on the joint surface to predict posttraumatic arthritis, compared with pressure measurements.

When analysing the data from our study, we interestingly found only a very small increase in DE in the setup with the current tolerance limit of a horizontal 2 mm gap in lateral tibial split fractures. One explanation for this could be the femoral roll back between the femur and meniscus, so the tibial plateau fracture hardly affects the friction: The meniscus has four properties, in our opinion mediating the compensation of the fracture. First, the meniscus makes the joint more congruent [30], so the small gap is equalized. Second, the largest distance in the fracture line is covered by the meniscus, caused by the anatomical situation in the lateral knee. The menisci occupy 70% of the contact area between femur and tibia and the lateral meniscus has a free periphery not attached to the capsule or tibia in its posterior half. Therefore the lateral meniscus gains more mobility compared with the medial meniscus [27]. Third, most of the load acting on the lateral tibia is translated through the meniscus, especially under higher loads [31], resulting in a further compensation of the fracture. Finally, the excellent lubrication function of the meniscus leads to an even lower friction coefficient than when directly articulating cartilage surfaces, which is rarely influenced by the gap in the tibial head. This is supported by the fact, that a higher friction coefficient could be measured after Meniscectomy [30].

These results are also supported by clinical trials: Stevens et al. found no significant difference in the WOMAC (Western Ontario and McMaster Universities Osteoarthritis Index) Score and the SF-36 Score (Short Form (36) Health Survey) between patients with anatomical and non-anatomical reduction in operatively treated tibial plateau fractures, but mentioning the small non-anatomic reduction group [32].

As key result of our study, we found a significant increase in DE when investigating the current tolerance limit of 2 mm step under vertical mismatch conditions. In a step-down condition the difference to native is almost doubled compared with the difference of DE in gap condition and when reducing the fracture in a step-up condition even tripled. However, there was no significant difference between the two conditions. Regarding this clear result with an obvious difference in DE a step up reduction should be avoided in daily practice.

When using other parameters to address the question of fragment position after reduction such as contact pressure or histological cartilage degeneration, similar findings were obtained: Bai et al. showed a significant increase in average contact pressure in rising step conditions (0–6 mm) in the lateral compartment in tibial plateau fractures. They described a significant increase in pressure even at a 1 mm step in 0° flexion [10]. These findings corroborate a sensitive change in the parameters regarding step condition. Histological cartilage degeneration is also induced by step conditions as Goetz et al. could demonstrate in an animal study. They compared anatomic reduction with a vertical 2 mm mismatch reduction in distal tibial fractures and found a more severe cartilage degeneration in the 2 mm step group 12 weeks after fracture, using the Mankin-Score for histopathological classification of the severity of osteoarthritic lesions of cartilage [9]. Nevertheless, no significant differences were found in the investigation of other parameters (peak vertical force and vertical pulse) between 2 mm step group and anatomical reduction group in this animal model.

### Study limitations

The measurements in the in-vitro model are limited to the moment after surgery. Long time effects like cartilage repair with fibroblasts [33] and the the bony fracture healing might reduce the DE and lead to different results. Especially in a non-anatomical reduction, the biomechanical and clinical outcomes are probably better than the results of DE in our study suggest, due to the mentioned healing effects.

To perform the study, only a limited number of specimens were available and we had to measure several conditions on the same joint. Therefore, statistical power of DE is limited and the tests previously made on the same specimen may affect the result.

Moreover, the necessary high load combined with the joint movement can - as in two specimens in our study - induce specimen failure. While our experimental setup can offer measurement conditions very close to the ones encountered in the native joint, when compared with other studies using static loads [10], we could only measure a reduced amount of conditions. Due to the biomechanically demanding stress on the joint during testing, especially the step-up conditions of 2 mm led to screw loosening and further fractures.

## Conclusion

We established a 3D kinematic test system that allows measurement of DE in human knee joints. When measuring DE in LTSF, small horizontal gaps with 2 mm in the lateral tibial plateau did not lead to a strong increase in DE. Interestingly, vertical gaps of the same size lead to a statistically significant and most probably clinically relevant increase in DE, with the step-up condition leading to the worst DE values. Based on our biomechanical findings we suggest to avoid especially vertical step conditions in the daily work in the operating theatre when reducing tibial head fractures. Horizontal gap conditions can be handled a bit more generously.

## Data Availability

The datasets used and/or analysed during the current study are available from the corresponding author on reasonable request.

## Abbreviations

DE: Dissipated energy
LTSF: Lateral tibial split fractures
SF-36 Score: Short Form (36) Health Survey
WOMAC: Western Ontario and McMaster Universities Osteoarthritis Index

## References

[1] Weimann A, Heinkele T, Herbort M, Schliemann B, Petersen W, Raschke MJ (2013). Minimally invasive reconstruction of lateral tibial plateau fractures using the jail technique: a biomechanical study. BMC Musculoskelet Disord.

[2] Lobenhoffer P, Schulze M, Gerich T, Lattermann C, Tscherne H (1999). Closed reduction/percutaneous fixation of tibial plateau fractures: arthroscopic versus fluoroscopic control of reduction. J Orthop Trauma.

[3] Papagelopoulos PJ, Partsinevelos AA, Themistocleous GS, Mavrogenis AF, Korres DS, Soucacos PN (2006). Complications after tibia plateau fracture surgery. Injury.

[4] Blokker CP, Rorabeck CH, Bourne RB (1984). Tibial plateau fractures. An analysis of the results of treatment in 60 patients. Clin Orthop Relat Res.

[5] Waddell JP, Johnston DW, Neidre A (1981). Fractures of the tibial plateau: a review of ninety-five patients and comparison of treatment methods. J Trauma.

[6] Solomon LB, Stevenson AW, Lee YC, Baird RP, Howie DW (2013). Posterolateral and anterolateral approaches to unicondylar posterolateral tibial plateau fractures: a comparative study. Injury.

[7] Prasad M, Yadav S, Sud A, Arora NC, Kumar N, Singh S (2013). Assessment of the role of fibular fixation in distal-third tibia-fibula fractures and its significance in decreasing malrotation and malalignment. Injury.

[8] Giannoudis PV, Tzioupis C, Papathanassopoulos A, Obakponovwe O, Roberts C (2010). Articular step-off and risk of post-traumatic osteoarthritis. Evidence Today Injury.

[9] Goetz JE, Fredericks D, Petersen E, Rudert MJ, Baer T, Swanson E (2015). A clinically realistic large animal model of intra-articular fracture that progresses to post-traumatic osteoarthritis. Osteoarthr Cartil.

[10] Bai B, Kummer FJ, Sala DA, Koval KJ, Wolinsky PR (2001). Effect of articular step-off and meniscectomy on joint alignment and contact pressures for fractures of the lateral tibial plateau. J Orthop Trauma.

[11] Isaac DI, Meyer EG, Haut RC (2008). Chondrocyte damage and contact pressures following impact on the rabbit tibiofemoral joint. J Biomech Eng.

[12] Walter C, Leichtle U, Lorenz A, Mittag F, Wulker N, Muller O (2013). Dissipated energy as a method to characterize the cartilage damage in large animal joints: an in vitro testing model. Med Eng Phys.

[13] Lorenz A, Rothstock S, Bobrowitsch E, Beck A, Gruhler G, Ipach I (2013). Cartilage surface characterization by frictional dissipated energy during axially loaded knee flexion--an in vitro sheep model. J Biomech.

[14] Bobrowitsch E, Lorenz A, Wulker N, Walter C (2014). Simulation of in vivo dynamics during robot assisted joint movement. Biomed Eng Online.

[15] Thomas C, Athanasiov A, Wullschleger M, Schuetz M (2009). Current concepts in tibial plateau fractures. Acta Chir Orthop Traumatol Cechoslov.

[16] Wilson DR, Feikes JD, Zavatsky AB, O'Connor JJ (2000). The components of passive knee movement are coupled to flexion angle. J Biomech.

[17] Wilson DR, Feikes JD, O'Connor JJ (1998). Ligaments and articular contact guide passive knee flexion. J Biomech.

[18] Schatzker J, McBroom R, Bruce D. The tibial plateau fracture. The Toronto experience 1968--1975. Clin Orthop Relat Res 1979(138):94–104. 445923

[19] Tscherne H, Lobenhoffer P (1993). Tibial plateau fractures. Management and expected results. Clin Orthop Relat Res.

[20] van den Bogert AJ, Reinschmidt C, Lundberg A (2008). Helical axes of skeletal knee joint motion during running. J Biomech.

[21] Corke P (2011). Robotics toolbox for MATLAB, release 9 [software].

[22] Tamer TM (2013). Hyaluronan and synovial joint: function, distribution and healing. Interdiscip Toxicol.

[23] Teeple E, Elsaid KA, Fleming BC, Jay GD, Aslani K, Crisco JJ (2008). Coefficients of friction, lubricin, and cartilage damage in the anterior cruciate ligament-deficient Guinea pig knee. J Orthop Res.

[24] Hui AY, McCarty WJ, Masuda K, Firestein GS, Sah RL (2012). A systems biology approach to synovial joint lubrication in health, injury, and disease. Wiley Interdiscip Rev Syst Biol Med.

[25] Hogel F, Hoffmann S, Panzer S, Wimber J, Buhren V, Augat P (2013). Biomechanical comparison of intramedullar versus extramedullar stabilization of intra-articular tibial plateau fractures. Arch Orthop Trauma Surg.

[26] Zhang W, Luo CF, Putnis S, Sun H, Zeng ZM, Zeng BF (2012). Biomechanical analysis of four different fixations for the posterolateral shearing tibial plateau fracture. Knee.

[27] Fukubayashi T, Kurosawa H (1980). The contact area and pressure distribution pattern of the knee. A study of normal and osteoarthrotic knee joints. Acta Orthop Scand.

[28] Trumble T, Verheyden J (2004). Remodeling of articular defects in an animal model. Clin Orthop Relat Res.

[29] Jay GD, Torres JR, Rhee DK, Helminen HJ, Hytinnen MM, Cha CJ (2007). Association between friction and wear in diarthrodial joints lacking lubricin. Arthritis Rheum.

[30] McCann L, Ingham E, Jin Z, Fisher J (2009). Influence of the meniscus on friction and degradation of cartilage in the natural knee joint. Osteoarthr Cartil.

[31] Walker PS, Erkman MJ (1975). The role of the menisci in force transmission across the knee. Clin Orthop Relat Res.

[32] Stevens DG, Beharry R, McKee MD, Waddell JP, Schemitsch EH (2001). The long-term functional outcome of operatively treated tibial plateau fractures. J Orthop Trauma.

[33] Mitchell N, Shepard N (1980). Healing of articular cartilage in intra-articular fractures in rabbits. Clin Orthop Relat Res.

